# A quasi-experimental assessment of the effectiveness of the Community Health Strategy on health outcomes in Kenya

**DOI:** 10.1186/1472-6963-14-S1-S3

**Published:** 2014-05-12

**Authors:** Rose Olayo, Charles Wafula, Evalyne Aseyo, Constantine Loum, Dan Kaseje

**Affiliations:** 1 Department of Community Development and Health Promotion, Tropical Institute of Community Health, Great Lakes University of Kisumu, P.O. Box 2224-40100, Kisumu; 2 Department of Health Services Management, Tropical Institute of Community Health, Great Lakes University of Kisumu, P.O. Box 2224-40100, Kisumu; 3 Department of Measurements, Tropical Institute of Community Health, Great Lakes University of Kisumu, P.O. Box 2224-40100, Kisumu

**Keywords:** Community health strategy, community dialogue, quasi-experimental design, community health workers, health outcomes, stratégie en santé communautaire, dialogue communautaire, concept quasi expérimental, agents de santé communautaire, santé

## Abstract

**Background:**

Despite focused health policies and reform agenda, Kenya has challenges in improving households’ situation in poverty and ill health; interventions to address the Millennium Development Goals in maternal and child health, such as focused antenatal care and immunization of children, are yet to achieve success. Research has shown that addressing the demand side is critical in improving health outcomes. This paper presents a model for health systems performance improvement using a strategy that bridges the interface between the community and the health system.

**Methods:**

The study employed quasi-experimental design, using pre- and post-intervention surveys in intervention and control sites. The intervention was the implementation of all components of the Kenyan Community Health Strategy, guided by policy. The two year intervention (2011 and 2012) saw the strategy introduced to selected district health management teams, service providers, and communities through a series of three-day training workshops that were held three times during the intervention period.

Baseline and endline surveys were conducted in intervention and control sites where community unit assessment was undertaken to determine the status of health service utilization before and after the intervention. A community health unit consists of 1000 households, a population of about 5000, served by trained community health workers, each supporting about 20 to 50 households. Data was organized and analyzed using Excel, SPSS, Epi info, Stata Cal, and SAS.

**Results:**

A number of health indicators, such as health facility delivery, antenatal care, water treatment, latrine use, and insecticide treated nets, improved in the intervention sites compared to non-interventions sites. The difference between intervention and control sites was statistically significant (p<0.0001) for antenatal care, health facility delivery, water treatment, latrine use, use of insecticide treated nets, presence of clinic card, and measles vaccination. Degree of improvement across the various indicators measured differed by socio-demographic contexts. The changes were greatest in the rural agrarian sites, compared to peri-urban and nomadic sites.

**Conclusion:**

The study showed that most of the components of the strategy were implemented and sustained in different socio-demographic contexts, while participatory community planning based on household information drives improvement of health indicators.

## Abstract (fr)

**Résumé:**

**Contexte**

Malgré des politiques de santé ciblées et un programme de réformes, le Kenya éprouve de la difficulté à améliorer la situation économique et sanitaire des ménages. Les interventions menées pour atteindre les objectifs du Millénaire pour le développement en matière de santé maternelle et infantile, comme des soins prénataux ciblés et la vaccination des enfants, n’ont pas encore donné les résultats escomptés. La recherche a révélé que, pour améliorer l’état de santé, il est crucial de prendre en compte la demande. Notre article présente un modèle d’amélioration du rendement des systèmes de santé fondé sur une stratégie qui relie les systèmes de santé communautaire et institutionnel.

**Méthodes**

L’étude repose sur une conception quasi expérimentale qui comprend des enquêtes réalisées avant et après l’intervention dans des sites sujets et témoins. L’intervention consistait à mettre en oeuvre toutes les composantes de la stratégie en santé communautaire du Kenya, en suivant des politiques. L’intervention a été lancée au sein de certains fournisseurs de services, équipes de gestion et collectivités et a pris la forme d’une série d’ateliers de formation s’étalant sur trois jours tenus trois fois pendant les deux années qu’a duré l’intervention (2011 et 2012).

Les enquêtes réalisées avant et après l’intervention ont été menées dans les sites sujets et témoins. On a effectué une évaluation de l’unité de santé communautaire pour déterminer le taux d’utilisation des services de santé avant et après l’intervention. Une unité de santé communautaire est constituée de 1 000 ménages, soit environ 5 000 personnes, servis par des travailleurs en santé communautaire formés qui travaillent auprès de 20 à 50 ménages chacun. Les données ont été organisées et analysées au moyen de divers outils : Excel, SPPS, Epi info, Stata Cal et SAS.

Certains indicateurs de santé se sont améliorés dans les sites sujets, comme la prestation de services dans les établissements de santé, les soins prénataux, le traitement de l’eau, l’utilisation des latrines et de moustiquaires traitées aux insecticides. En ce qui a trait aux soins prénataux, à la prestation de services dans les établissements de santé, au traitement de l’eau, à l’utilisation des latrines et de moustiquaires traitées aux insecticides, à la présence de cartes de clinique et à la vaccination contre la rougeole, la différence observée entre les sites sujets et les sites témoins était considérable sur le plan statistique (<0.0001). Le taux d’amélioration des différents indicateurs mesurés variait en fonction du contexte sociodémographique. Les indicateurs fluctuaient davantage dans les milieux ruraux agricoles que dans les milieux périurbains ou nomades.

L’étude a montré que la plupart des composantes de la stratégie ont été mises en oeuvre et maintenues dans différents contextes sociodémographiques, et la planification communautaire participative fondée sur les données sur les ménages améliore la santé globale.

## Introduction

Globally, the number of child deaths decreased from 12.5 million in 1990 to 8.8 million in 2008 [1]. Neonatal deaths accounted for about one third of childhood deaths and are linked closely to slow progress in the reduction of maternal mortality. It is estimated that globally about 342 900 maternal deaths occur each year [2]. There has been no substantial change in maternal mortality in sub-Saharan Africa over the past ten years and therefore progress towards improving child deaths has remained slow in this region [3].

The high maternal and newborn mortality in sub-Saharan Africa is related to unsafe maternal and newborn health (MNH) practices [3]. Puerperal infections remain a major cause of maternal mortality, partly due to poorly observed rules of cleanliness and an unhygienic delivery environment. Most newborn deaths occur during the first week of life as a result of sepsis, birth asphyxia, birth injuries, complications of prematurity, low birth weight, and birth defects [4]. It is also tragic that the recurrence of adverse perinatal outcomes in many developing countries is still prevalent [5]; that is, a woman whose first pregnancy ends in stillbirth or is followed by the death of the neonate is at increased risk of experiencing the same outcomes in her second pregnancy; this situation is common in these communities.

The maternal and neonatal health trends in Kenya are similar to other sub-Saharan African countries. In Kenya, the maternal mortality ratio is estimated to be 488 women per 100 000 live births, up from 443 women per 100 000 live births in 2003 [6]. Maternal deaths represent about 15% of all deaths of women aged 15 to 49 years [7]. Between 2003 and 2008, the under-five and infant mortality rates declined by 36% and 32%, respectively.

Experience over the past decade has shown that to improve maternal and newborn health and reduce morbidity and mortality, efforts should focus on building capacities at individual, family, and community levels to ensure appropriate self-care, prevention, and care-seeking behavior [8].

This assertion is in line with a global study reported in the Lancet by a team of researchers [9]; they found effective reductions in maternal and child mortality. They reanalyzed coverage data from 312 nationally representative household surveys done between 1990 and 2011 in 69 countries including Kenya. Their observation was that substantial reduction in child deaths are possible, but only if intensified efforts to achieve intervention coverage are successfully implemented.

In Africa, the majority of deaths (60%) occur at home without any contact with the health system [10]; they also recognize the role of Kenya’s Ministry of Public Health and Sanitation (MOPHS) through its National Health Sector Strategic Plan II (NHSSP II) in seeking to improve health outcomes through promotion of individual and community health. The purpose of the NHSSP II was to strengthen health services through several strategies, one of which was the Community Health Strategy (CHS) [10].

At the national level in Kenya, the CHS’ major goal is to enhance community access to health care as a way of improving individual productivity to reduce poverty, hunger, and reduce child and maternal deaths, as well as improve education, (see Community Strategy Implementation Guidelines, Ministry of Health, Kenya); these guidelines offer the detailed operations of the strategy which is to be embraced nationally.

Primary decisions and actions that influence the health outcomes of a community are made at the household level [11]. It is noted that addressing the supply side of health care is important but not sufficient in improving the health status of a population; however addressing the demand side is critical in improving health outcomes [12]. Community based health workers have been found to be effective in improving the health status of the communities, therefore increasing the demand of services [13,14].

It is important to negotiate with households and communities as equal partners in health care, giving them a chance to make decisions on the way care is delivered, to include their participation and gain their confidence in the health system [15]. Health systems research cannot be expected to solve all of the problems facing the health system. However, it does have a central role to play. A more solid knowledge and evidence base would help inform the challenges associated with the uptake of health services.

Although there are studies that reasonably capture the scope of issues that are important, they do not reveal much about the socio-demographic contexts, specifically across the rural agrarian, peri-urban, and nomadic contexts. It is therefore important to establish the fundamental and empirical evidence to determine the uptake and effectiveness of the Community Health Strategy in different socio-demographic contexts in Kenya.

It is for this reason that our study sought to investigate the uptake and effectiveness of the Kenyan Community Health Strategy in different socio-demographic contexts. The strategy engages both the service delivery system and the communities served in an iterative process of dialogue, informed by community based information systems, to synergize efforts and motivate both the service consumers and providers towards health status improvement. Little is known about the uptake and effectiveness of the Community Health Strategy and how this could contribute to scaling up of health services in the face of urgent public health problems in Kenya. The main objective of the study was to find out whether the use of structured evidence based information sharing and dialogue leading to planning and action would facilitate improvement in health outcomes in different socio demographic contexts in Kenya.

The main research question the study aimed to address was:

What is the uptake and effectiveness of the Community Health Strategy in different socio-demographic contexts?

The study objectives were:

1. To identify and assess the components of Community Health Strategy that have been implemented and sustained in nomadic, peri-urban, and rural contexts in Kenya.

2. To compare the uptake of elements of the Community Health Strategy in nomadic, peri-urban, and rural agrarian contexts in Kenya.

3. To assess the influence of the implementation of the Community Health Strategy on health outcomes in nomadic, peri-urban, and rural agrarian contexts in Kenya.

## Methodology

The study was a quasi-experimental design, using pre- and post-intervention surveys, in intervention and control sites. The intervention in this study was based on implementation of all components of the Community Health Strategy as per the policy implementation guidelines. The study engaged the consumers, policy makers, community and the health system managers in the processes of research to improve health service utilization.

A stakeholder analysis was done to ensure the participation of all stakeholders and have a consensus on the study framework and methodology. It involved the participation of health management teams both at the province and district levels, the community and local government administration. The study sites were identified and selected during the stakeholder analysis process.

The selection of the intervention sites was based on the willingness of the communities to participate. In comparison, a control matched site was identified for assessments. For the control sites, factors such as geographical location, service coverage, including level of health facility, was considered. Baseline surveys were conducted to confirm statistical congruence between intervention and control sites in terms of socio-demographic factors. A total of eight community health units and eight health dispensaries were involved as intervention sites. The rural agrarian study site consisted of four community health units and four health facilities, while peri-urban had two community health units and two health facilities, and two community units and health facilities for the nomadic site. A community health unit was defined by the population coverage of 5000 population and with about 1000 households. An initial pre-intervention assessment was conducted in early 2010 while post-intervention observation took place two years later, in 2012.

**Figure 1:**
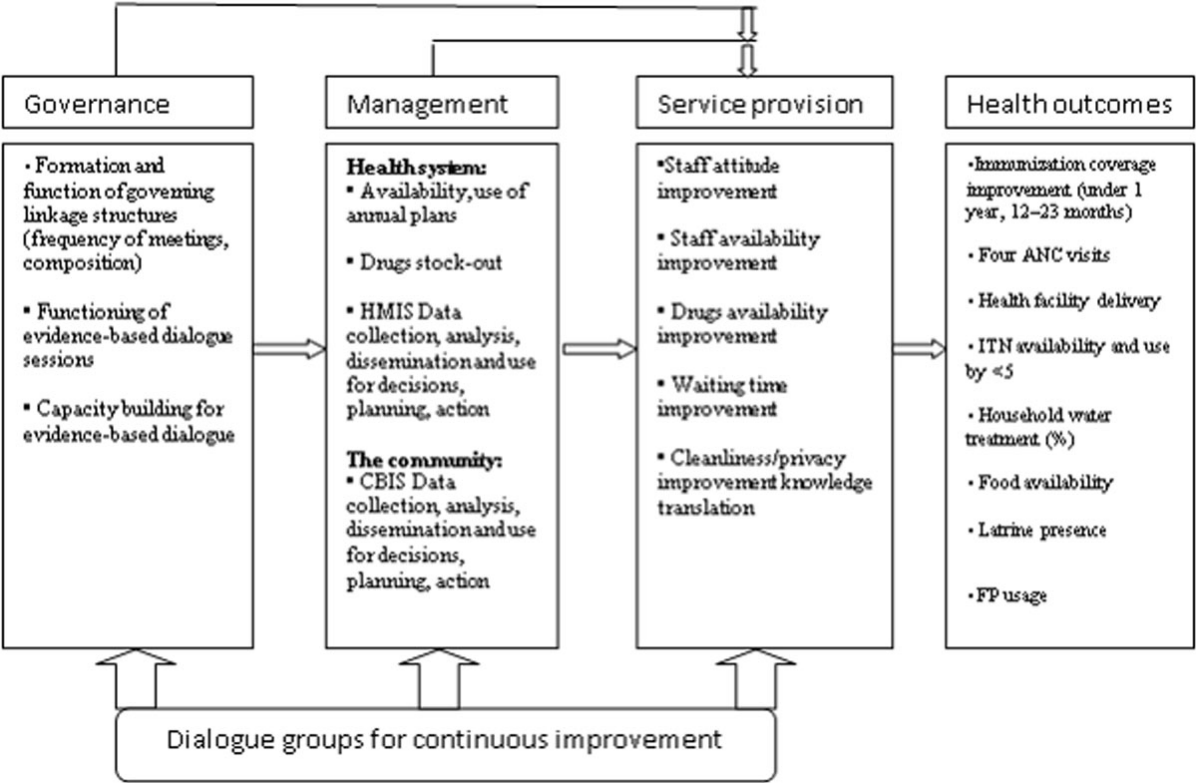
Logical framework of the complex intervention Adapted from Kaseje *et al*, 2011

The study took two years and was introduced to the selected health management teams at the district level, service providers, and the community. For consistency in the implementation process, three day trainings were held in three phases during the intervention period. The first phase involved building the capacity of the implementers with the necessary skills to implement the intervention. The research team members participated in all trainings together with the district health team members to ensure consistency in implementation. The conceptual framework in Figure 1 demonstrates the linkage between the community and the health system in governance, management, and service provision leading to health outcomes. The key elements of the intervention package included (see Figure 1):

• The formation of committees at the community and health facility levels as governance/linkage structures.

• The identification and training of community health workers to support households in health improvement initiatives, as well as to maintain the village register, and facilitate dialogue at the household level.

• The community health workers are lay volunteer health workers covering 20 to 50 households within their own villages where they reside. They volunteer their services to work within their neighborhoods.

The key elements of the intervention also involved the identification, training and deployment of community health extension workers (CHEWs) for each community unit as the facilitator of dialogue at the community level and supporter of CHWs. They were also the maintainer of the Community Based Health Information System. The CHEW is responsible for supervision of the CHWs within a sub-location. CHEWs are professional health workers employed by the health system, living and working in the community.

The process also involved the establishment of village registers of all households to provide community based information, including all health status aspects targeted for improvement. The information collected in the household registers was updated every six months by the community health worker to monitor change in health seeking behavior among the household members. The information from the registers was analyzed and displayed on chalk boards within the sub-location. This would lead to timeliness of analysis, dissemination, and utilization of Health Management Information System (HMIS) data. Once collected at the sub-location level, reports were submitted to the district level for electronic processing. The intervention facilitated manual analysis of relevant health facility data for posting on chalk boards at the sub-location level.

Client satisfaction interviews using questionnaires were conducted and analyzed every six months to gauge the level of service satisfaction. Dialogue sessions were held based on data from the community and health facilities depicting the current situation regarding elements targeted for improvement. The dialogue sessions were held on a monthly and quarterly basis at household and community levels, respectively, and every six months at a health facility and the sub-district levels. The dialogue sessions were facilitated by CHWs during home visits and by CHEWs at general community meetings, while the health facility staff facilitated dialogue at the management committee meetings and the District Health Management Teams (DHMTs) facilitated at sub-district health stakeholder forums. At this level timing was based on the district reporting cycle.

The dialogue process was attended by managers, service providers, and community representatives from community units representing defined constituencies in their community. The dialogue process involved displaying the data from the health facilities and from the community chalkboard to clearly depict the current situation in the community. This was then followed by discussion towards consensus building regarding the data presented and what was not acceptable and what needed improvement. Action towards improvement was agreed on and a plan of action developed, with targets to be achieved before the next dialogue session. Since the sessions at the community and sub-district levels were as large as 50 people or more, the action planning stage of the process was undertaken in groups of 8 to 12 participants (usually the community health committee).

**Figure 2:**
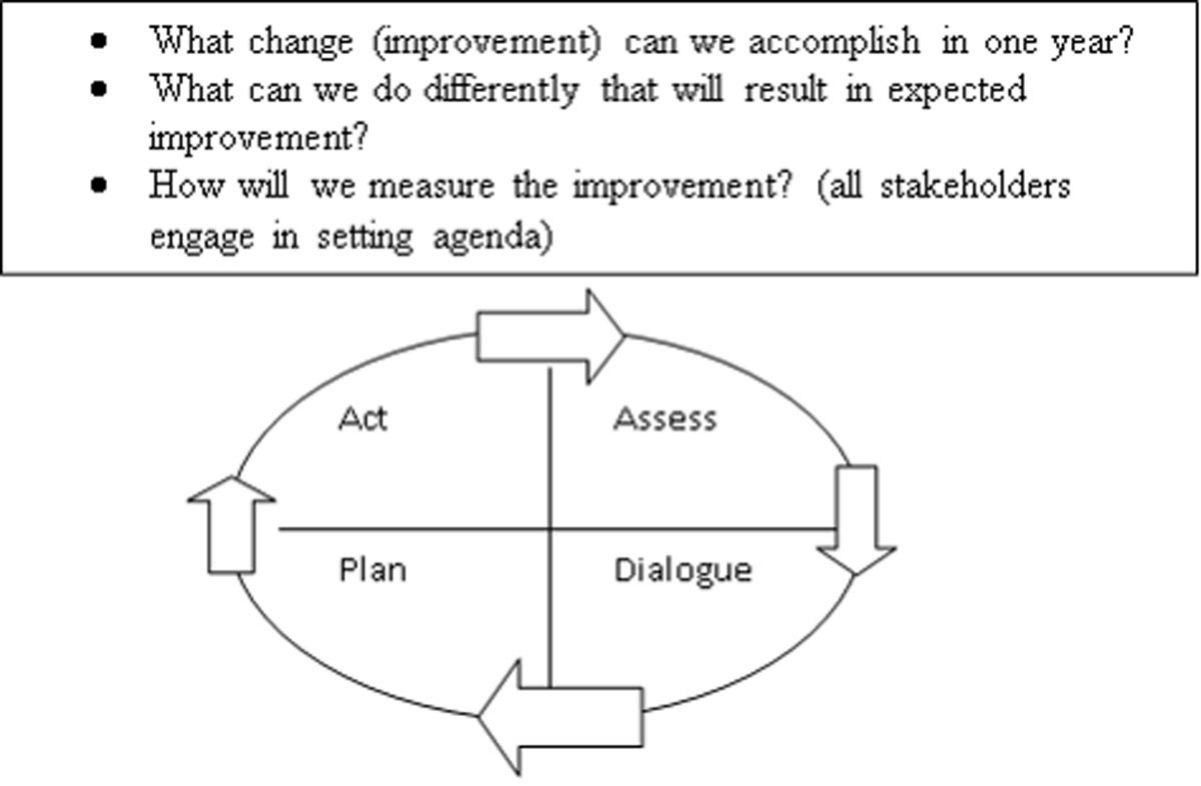
Dialogue to Drive Planning, Action Adapted from: F *et al*, 2011

Depending on the level, the timeframe for the dialogue session varied from about an hour at the community level and much longer at the sub-district level. The dialogue process took place at household, community unit, health centre, and sub-district levels, based on issues emerging from the data gathered and analyzed at every level. The data utilized for dialogue came from household registers interpreted by CHWs, community unit summaries prepared by CHEWs, and quarterly reports. The time frame was flexible based on level and issues for discussion. The participation also varied with the level. The participants increased with the level, from household to sub-district (Figure 2).

### Ethical considerations

Ethical review was obtained at four levels. The study protocol was first presented to the Great Lakes University of Kisumu Ethics Review Board, which is the lead research institute, and later to Moi University Ethics Review Board. Both Ethics Review Boards granted the clearance for the study to be undertaken. Further permission and clearance was obtained from the Kenya Ministry of Health (then MOPHS) through the DHMTs within the study districts of Butere, Mumias, Kisumu, and Garissa. Informed consent was obtained from individuals who participated in the face-to-face interviews after having been appropriately informed of the purpose of the study.

Participation in the study was voluntary and there were no incentives granted in return, except to the extent that the process led to improvement in health indicators within intervention sites. Confidentiality of data was maintained in several ways. Firstly, data collection was done by research assistants who had been trained on code of conduct and confidentiality of data. The research assistants signed a code of conduct and terms of reference stipulating the standards expected. Data processing into electronic versions was also done by trained data clerks who had signed terms of reference contracts with clear stipulation of expected standards of confidentiality. Both electronic and manual data were kept under lock and key by a senior research officer and supervision of the Principal Investigator (PI). The PI has the sole responsibility of approving access to the data so stored.

### Study setting

The study was undertaken in four units in rural agrarian, two in peri-urban, and two in nomadic sites, which were purposively selected from three different contexts (social, economic, and ecological) in Kenya. The four rural agrarian Community Health Units (CHUs) included in the study were situated in Butere district, Western Kenya while peri-urban and nomadic districts were situated in Kisumu and Garissa, respectively. All selected CHUs were part of the Community Health Strategy scale-up partnership districts between the Ministry of Public Health and Sanitation and the Great Lakes University of Kisumu (GLUK). They comprised CHUs that were in the process of being formed and required to go through the complete cycle of establishment, operation, and sustainability. In the Kenyan Community Health Strategy, local populations of 5 000 were served by a community health unit served by 30 CHWs, each providing health services to 20 to 50 households. However, CHWs in the peri-urban area served up to 100 households due to high density of households.

Study sites were purposively selected based on readiness to launch the Community Health Strategy. The control sites were selected from neighboring districts, which were matched by geographical location, ethnicity, means of earning livelihoods and economic status. Four, two, and two health facilities were selected in Butere, Nyalenda, and Garissa, respectively. The same numbers of health facilities were selected in the respective control sites. All CHUs served by these health facilities were included, as were all households within the selected units. For baseline and endline surveys, a 20% sample was randomly selected for the surveys in both control and intervention sites.

### The assessments

Data for assessment was collected on the implementation level of elements of the Community Health Strategy at the unit and health service utilization level. This was done on a biannual basis and comprised collection of data on:

1. Functionality of community health committee, based on number of training days, frequency of meetings verified by a copy of minutes, representation on link health facility management committee;

2. Functionality of the community health workers, based on number of training days, frequency of CHW meetings, village representation, attendance at CHW meetings, and frequency of household visits.

3. CHEW support to the CHU, measured by the number of supportive visits to CHWs, and number of refresher trainings for CHWs.

4. Community based health information system functionality, measured by the coverage of households registered in the unit, frequency of household register updates, and number of monthly chalk board updates.

5. Frequency of community dialogue days held, presence of reports, and attendance level at the dialogue days.

6. Frequency of community action days conducted by the unit, presence of reports, and attendance level at the action days.

Health services utilization levels were assessed at the beginning and end of the study through community surveys. The community health unit assessment was undertaken by trained research assistants only in the intervention sites every six months. Data on functionality of community health units was captured manually through interviews carried out by trained research assistants.

### Survey

Baseline and endline surveys were conducted before and after the intervention to determine the status of health service utilization. These surveys were conducted in both the study and control sites. A baseline tool was developed to cover household demographic characteristics, individual health services utilization, morbidity among household members, and mortality in the household over the six months preceding the survey. Indicators measured included health facility delivery, antenatal care, presence of clinic cards, immunization coverage, vitamin A supplement, use of modern family planning methods, use of treated nets, water treatment, latrine use, and food availability.

The tool was pre-tested to ensure validity and reliability of the data that would be collected. It was administered by trained research assistants who were supervised by senior researchers. Face-to-face administration of the tool to household heads took on average 20 minutes.

### Analysis

Analysis was undertaken using five software (Excel, SPSS, Epi info, Stata Cal, SAS). The first level of data analysis was undertaken of baseline and endline data collected in October 2010 and December 2012, respectively, to describe the intervention and outcome variables for each of the study sites. The comparison analysis was done both for individual CHUs and aggregated by districts, then a district by district comparison was done. Combined data from all sites was also analyzed descriptively. Bivariate analysis was undertaken to determine relationships between the Community Health Strategy intervention and health outcomes by comparing baseline and endline outcome variables in intervention and control sites. Chi square test was used to determine the significance of differences in dependent outcome proportions (pre-post, and intervention versus control), using the Community Health Strategy elements as independent variables. A p-value of <0.05 was considered statistically significant.

A binary logistic regression analysis was used to assess the effect of Community Health Strategy on health outcomes. In the model, we created binary variables; study arm (intervention=1, control =0), post (endline survey=1, baseline survey=0), and an interaction factor coded as 1 (if study arm=1 and endline=1) and 0 otherwise. These were added in the model as predictor variables while the binary health outcome variables included: attending antenatal care four times or more, health facility delivery, and availability of immunization card, presence of a latrine, use of insecticide treated nets, use of treated water, pentavalent 1, pentavalent 3, and availability of food in the household.

Each outcome had its own regression model. In order to interpret the results from this regression model, we considered that we were testing the null hypothesis that differences in proportions of health outcomes in the baseline and endline surveys in control sites was equal to differences in the baseline and endline surveys in the intervention sites. The interaction factor helped us to control for those households that participated in both baseline and endline surveys. To test if the intervention worked, we tested the coefficient of interaction factor in zero (which is the odds ratio of the co-efficient of the interaction factors of those who were in the study arm and in the post test survey). If the odds ratio and 95% confidence interval (CI) of this factor was >1 then the intervention worked, if OR<1 then control did better than intervention sites, if the interval was 0 then there was no effect.

The comparative pooled analysis method was used to combine data from the six sites. A Poisson regression model was used to assess the effect of Community Health Unit components on the health service utilization outcomes. The Poisson regression model was used because the outcome of interest was considered as count data and the data was analyzed on population level rather than household level.

## Results

The results from baseline surveys indicate comparability of basic characteristics at intervention and matched control sites. At the nomadic sites, antenatal coverage, health facility delivery, water and sanitations coverage were similar, essentially worse than rural agrarian and peri-urban sites. Similarly, the urban intervention and control sites had similar coverage rates, both being much better than rural and nomadic sites. Coverage rates at rural agrarian sites were worse at intervention than control sites at baseline. Thus baseline data confirmed suitability of control sites to permit post-intervention comparisons, with regard to effectiveness of the intervention in influencing health outcomes.

### Governing structures

The study found that the Health Facility Committees existed but were dormant, while Community Health Committees were present and functional at all intervention sites.

### Community health committee

As shown on table 1, Community Health Committees existed and were actively involved in implementation of the Community Health Strategy, as demonstrated by the frequency of meetings held and availability of minutes, action plans, and implementation plans.

**Table 1:** Community health committee functionality in the rural site (Butere)

		Mutoma	Bubala	Shibembe	Shitari
	
CU Component		2012	2012	2012	2012
**CHC functionality**					
1	Existence	Yes	Yes	Yes	Yes
2	Membership	12	12	14	12
3	Composition	4	4	5	3
	Representativeness (M/Fin %)	33/67	42/58	79/21	67/33
4	Linkage to HF	Yes	Yes	Yes	Yes
5	HF representation	Yes	Yes	No	Yes
6	No. of meetings	2	4	12	3
7	Copy of minutes	Yes	Yes	Yes	Yes
	Actions (do they end with?)	Yes	Yes	Yes	Yes

CBHIS: community based health information system; CHC: community health committee; CU: community unit; HF: health facility

### Community based workers

The study found that CHWs in all communities were available at baseline and endline and they had been trained on various topics as stipulated by the Community Health Strategy guidelines. Their key roles included household registration and update every six months, monthly household visits, active case finding mainly in households with pregnant women and under-five children, and referrals of pregnant women to the clinics for antenatal visits. The study further found that CHEWs were not present at baseline but present at endline in the intervention sites.

### Community based health information system

As evident on table 2, the Community Based Health Information system was functional and this could be easily identified from the chalkboards that were updated on a monthly basis and topics for dialogue drawn from the chalkboards.

**Table 2:** Community based health information system functionality

		Mutoma	Bubala	Shibembe	Shitari
	
CU Component		2012	2012	2012	2012
**CBHIS**					
1	% population coverage	6252 (100%)	3422 (100%)	6040 (100%)	8824 (100%)
2	No hh covered	1450	847	1472	2150
3	No. of updates	2	2	2	2
4	Chalkboard presence	yes	yes	yes	no
5	Frequency of updates	monthly	monthly	monthly	n/a
6	When last updated	Dec	Nov	Dec	n/a

CBHIS: community based health information system; CU: community unit

### Evidence-based dialogue

**Figure 3:**
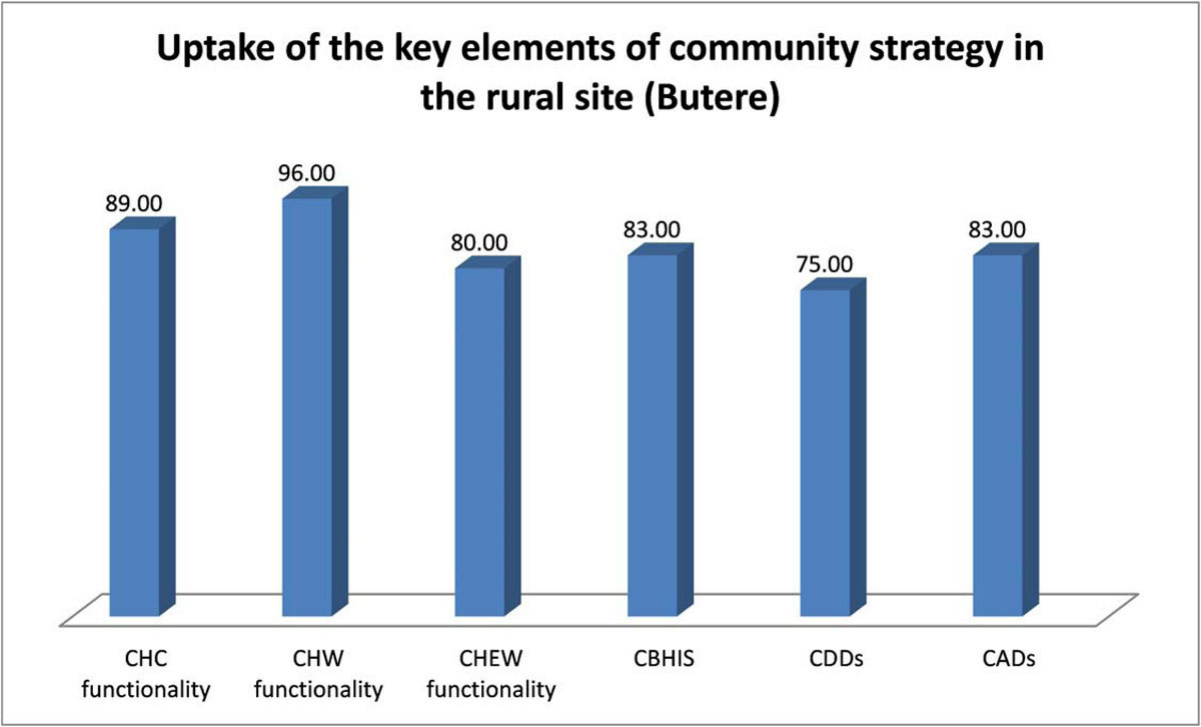
Uptake of the key elements of community strategy in the rural site (Butere) CHC: community health committee; CHW: community health workers; CHEW: community health extension worker; CBHIS: community based health information system; CDDs: community dialogue days; CADs: community action days

Dialogue was the major component of the Community Health Strategy based on evidence from the community dialogue days, which attracted more participants as compared to community action days (100 compared to 17). Key topics for community dialogue days were drawn from the chalkboard, i.e. immunization, solid waste management, water treatment, family planning, and antenatal care. The outcome of the dialogue led to an action day where the entire community took action to implement the key actions from the dialogue day. Such key actions implemented on action day included CHWs identifying children who were not immunized from their household records and referring them to the health facility attached to the CHU for immunization. Figure 3 shows that the key elements of the Community Health Strategy were taken up well, particularly in the rural site.

### Health outcomes

Tables 3, 4, and 5 show that there was greater improvement in health indicators in the intervention sites as compared to non-interventions sites in the rural, peri-urban, and nomadic contexts. However, changes in indicators measured were different for different socio-demographic contexts. Of all the nine key indicators measured, rural context had the greatest improvement as eight out of nine indicators improved, as compared to nomadic and peri-urban contexts where five and six indicators measured improved, respectively.

**Table 3:** Health outcomes between intervention and control sites at baseline and endlines in the rural site (Butere and Mumias)

	Mumias (Control site)				Butere (intervention site)			
Health indicator	Baseline	Endline	Change	*p*-value	Baseline	Endline	Change	*p*-value

ANC	66.3	56.0	-10.3	<0.0001	47.7	72.0	24.2	<0.0001
HFD	49.5	46.3	-3.2	0.173	41.1	47.2	6.1	<0.0001
Water	31.6	81.1	49.5	<0.0001	37.1	93.2	56.0	<0.0001
Latrine	91.1	96.0	4.9	<0.0001	80.5	94.5	14.0	<0.0001
Card	85.1	86.3	1.2	0.486	91.0	89.3	-1.7	0.025
ITN	58.7	94.5	35.7	<0.0001	61.4	91.4	29.9	<0.0001
Penta 1	92.0	92.1	0.1	0.95	91.4	91.7	0.3	0.732
Penta 3	87.5	88.6	1.1	0.46	89.5	90.1	0.7	0.417
Food	62.3	81.3	19.0	<0.0001	36.0	56.4	20.3	<0.0001

ANC: antenatal care; HFD: health facility delivery; ITN: insecticide treated net

**Table 4:** Comparison of Health Outcomes between Intervention & Control Sites at baselines/endlines in the Peri urban site (Nyalenda and Obunga)

		Baseline		Endline		% change	
		
	Int/Con	Int	Con	Int	Con	Int	Con
ANC	Nyalenda/Obunga	82.84	85.96	76.26	48.94	-6.58	-37.02
HFD	Nyalenda/Obunga	82.84	73.44	79.69	67.27	-3.14	-6.17
Water treatment	Nyalenda/Obunga	62.66	65.42	65.53	61.63	2.87	-3.78
Latrine	Nyalenda/Obunga	79.87	86.22	89.76	89.39	9.89	3.17
Clinic Card	Nyalenda/Obunga	25.70	88.71	77.42	68.21	51.72	-20.49
ITN	Nyalenda/Obunga	75.49	82.05	89.30	97.32	13.81	15.26
Penta 1	Nyalenda/Obunga	89.69	95.89	88.68	89.25	-1.01	-6.65
Penta 3	Nyalenda/Obunga	88.80	92.42	49.03	86.69	-39.78	-5.73

ANC: antenatal care; HFD: health facility delivery; ITN: insecticide treated net; Int: intervention; Con: control: Penta - Pentavalent is a vaccine, administered to children in a three-dose schedule (Penta 1, Penta 2 and Penta 3), which offers protection against five diseases: diphtheria-tetanus-pertussis (DTP), hepatitis B, and *Haemophilius influenzae* type b

**Table 5:** Comparison of health outcomes in the intervention site at baseline and endline at Garissa

	Garissa baseline 2011				Garissa endline 2012				P value
	**Yes**	**No**	**Total**	**%**	**Yes**	**No**	**Total**	**%**	

ANC	289	241	530	54.53	396	359	755	52.45	0.462
HFD	160	370	530	30.19	274	486	760	36.05	0.028
Latrine	331	399	730	45.34	1904	1988	3892	48.92	0.076
Immunization Card	348	129	477	72.96	407	319	726	56.06	<0.0001
ITN use	423	309	732	57.79	2812	1080	3892	72.25	<0.0001
MUAC_Normal	433	82	515	84.08	392	34	426	92.02	0.0002
Penta 2	377	76	453	83.22	660	42	702	94.02	<0.0001
Penta 3	356	99	455	78.24	627	116	743	84.39	0.007

ANC: antenatal care; HFD: health facility delivery; ITN: insecticide treated net; MUAC: Mid-upper arm circumference; Penta - Pentavalent is a vaccine, administered to children in a three-dose schedule (Penta 1, Penta 2 and Penta 3), which offers protection against five diseases: diphtheria-tetanus-pertussis (DTP), hepatitis B, *Haemophilius influenzae* type b; Normal MUAC -MUAC is the assessment of nutritional status using the circumference of the left upper arm, measured at the mid-point between the tip of the shoulder and the tip of the elbow. In children, MUAC over 135mm (13.5cm), green colour, indicates that the child is well nourished, i.e. normal MUAC

**Figure 4:**
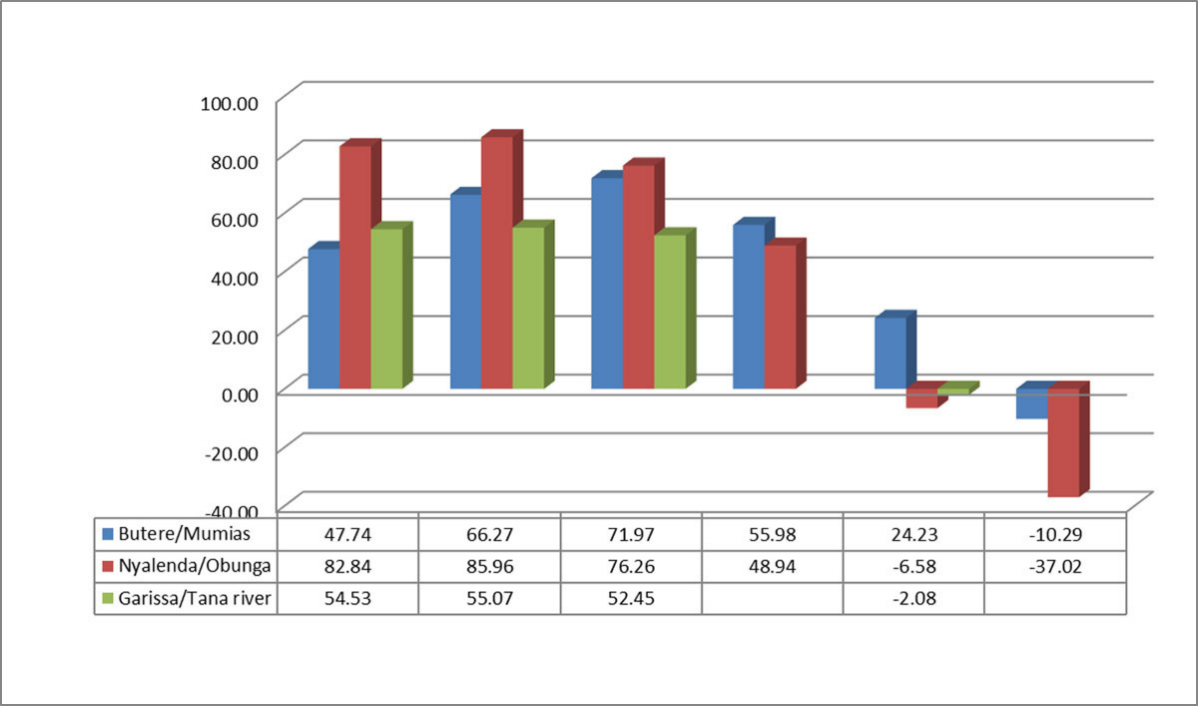
Comparison of antenatal care by socio-demographic context at baseline and endline

**Figure 5:**
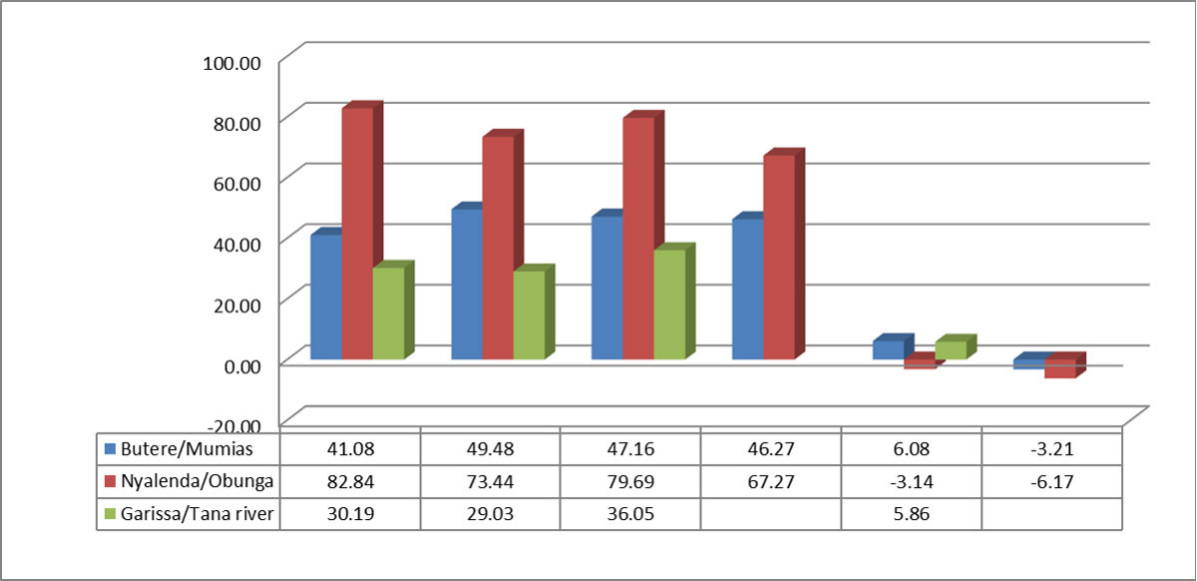
Comparison of health facility delivery by socio-demographic context at baseline and endline

Similarly, figures 4 and 5 show there was more improvement in health indicators in the intervention sites compared to non-interventions sites, and in the same vein, the change in indicators measured were different for different socio-demographic contexts. Only rural agrarian had a significant change in antenatal care in the intervention sites, as compared to the peri-urban and the nomadic sites. Whereas the number of women who delivered under skilled attendants improved in rural agrarian and nomadic settings, peri-urban had a negative change. The change was much more significant in the rural agrarian as compared to peri-urban and nomadic sites.

In general, the changes in the health outcomes between intervention and control sites were scientifically significant in most indicators such as health facility delivery, antenatal care visits, presence of clinic cards, measles vaccination, water treatment, and food availability as indicated in Table 6.

**Table 6:** Overall effect of CHS intervention on health outcomes, results from logistic regression

	Odds ratio	95% CI	P value
HFD	1.955	1.792-2.132	<.0001
ITN	1.115	0.881-1.410	0.3666
ANC	21.162	8.717-51.376	<.0001
Card	0.439	0.302-0.638	<.0001
Latrine	2.115	0.766-5.842	0.1484
Measles	1.144	1.011-1.296	0.0335
Penta1	0.662	0.328-1.337	0.25
Penta2	N/A	N/A	N/A
Penta3	1.073	0.612-1.882	0.8062
Water use	5.12	4.87-5.383	<.0001
Food	2.679	2.56-2.803	<.0001
Hand washing	0.035	0.026-0.047	<.0001

ANC: antenatal care; HFD: health facility delivery; ITN: insecticide treated net; Penta - Pentavalent is a vaccine, administered to children in a three-dose schedule (Penta 1, Penta 2 and Penta 3), which offers protection against five diseases: diphtheria-tetanus-pertussis (DTP), hepatitis B, *Haemophilius influenzae* type b

## Discussion

The results show that the Community Health Strategy is an effective approach to delivering community-based interventions. Importantly, in our study, there were significant changes in essential maternal and newborn care practices such as antenatal care attendance and skilled deliveries. The positive health outcomes documented by the study came about because household members had been provided with the necessary information to make healthy decisions to be able to respond to maternal and neonatal health needs, based on evidence provided by the community based health information system.

Our results are consistent with the results of others, who have demonstrated the benefits of focusing efforts on building capacities at the individual and family levels. Community based interventional studies in India and Guatemala, where neonatal and infant mortality rate reduced by 25% and 85%, respectively, provide additional support for targeted strategies at the community level [3]. The change in attitude by communities, based on evidence, facilitates behaviour change that improves pregnancy outcomes for women and health outcomes for newborns and infants. It also breaks inherent traditional practices to the benefit of future generations [4].

While not all indicators at the health outcome level improved consistently in our study, there were notable improvements across all three socio demographic contexts in latrine use, water treatment, and use of insecticide treated nets. While health facility deliveries improved significantly, these improvements were limited to the rural agrarian and nomadic contexts (in Butere and Garissa). The peri-urban site did not show significant improvement on this indicator.

It has been noted, however, that immunization coverage rates are also highly dependent on both supply and demand, such that facility-based initiatives or community-based initiatives may be insufficient, in and of themselves, to impact immunization coverage rates, as has been shown in previous reports pointing to the importance of supply and demand as critical to improved uptake [12].

Consistent with the aims of the Community Health Strategy, the model emphasized collection of relevant data to inform evidence-based planning and provided a forum for exchange of information between the providers and clients. Our results support recent research from South Asia where village communities were shown to be effective, through dialogue, in the reduction of maternal and perinatal mortality [16]. Similarly, a study undertaken in the Gambia that looked at how dialogue can be used to improve antenatal clinics based on opportunity for information, education, and communication, also showed that based on information, women were more able to make informed choices.

The HMIS generated from the facilities and the district health surveillance data were the key sources of information available for planning in the district, at baseline. At follow-up evaluation, the Community Based Health Information System had become more available, and recognized. It enhanced knowledge translation and information sharing. District level plans were better able to take advantage of information from the HMIS and Community Based Health Information System, which enhanced the evidence-based planning process, resulting in the development of realistic plans. However, the capacity for data management did not change much during the research period. Only the district offices had computers and personnel for data management.

Findings from these eight community health units illustrate the complex challenges faced by policy makers, program planners, and managers in establishing, maintaining, and sustaining effective and functional community health units in different socio demographic contexts. The implementation of the strategy and intensity was different in the different socio demographic contexts, given the complexities in the varied contexts.

### Study limitations

Our study has a number of important limitations. The most important of these was selection of intervention and control sites. Since the implementation of the Community Health Strategy was a government policy that was encouraged in all districts, it is possible that the districts that were moving ahead with implementation, hence selected as intervention sites, had other enabling factors that could influence health outcome improvement over and above the intervention. We attempted to reduce bias by matching the control to the intervention districts by geographical location, ethnicity, infrastructure and socio-cultural characteristics. However it was not possible to control for such factors as leadership and management effectiveness that would influence both the intervention and the health outcomes.

## Conclusions

The Community Health Strategy is playing a key role in the renewal of comprehensive primary health care in Kenya. It improves access to health care, and thus improves health outcomes. Involvement of the community in the planning procedures and implementation has started to attract levels of resource allocation to the lowest level of care, leading to equitable distribution of resources, using an integrated approach for multidimensional and multi-sectoral health programming.

The model in this study has shown that most of the components can be taken up and sustained with different interventions for different socio-demographic contexts. There is need to balance priorities on the uptake and elements of the Community Health Strategy in different socio-demographic contexts in Kenya. It is important that research agendas and questions address the needs of various contexts and that recommendations take into account the reality of different contexts including practicality, budget and service delivery issues. Consulting and working with policymakers and other key stakeholders will help ensure that research agendas are aligned to national problems and priorities.

In addition, interactions and engaging health policymakers and managers in the research process is a particularly effective way of communicating research findings and making use of the evidence from research. This is enhanced when researchers have reputations for producing credible evidence and have established strong and trusting relations with policymakers. However, it is also important to maintain communication with policymakers throughout the research process to provide updates and develop trusting relationships. The glaring gap between researchers and policy makers needs to be narrowed through a serious, ethical and honest rethink of the research prioritization process at national, regional, and local levels.

The Community Health Strategy ensures the community’s increased access to most health services. The model has shown that most of the components can be taken up and sustained, however it has been shown to differ by socio-demographic contexts. Importantly, this strategy has also led to improvement in health indicators, to the benefit of the community.

In reviewing the existing Community Health Strategy policy, it is crucial to review and modify the implementation based on the complexities surrounding the various socio-demographic contexts. This can be done by tailoring the elements of the strategy to socio-demographic contexts. The number of households allocated to CHWs should be based on population density. Peri-urban CHUs should be formed at the village level rather than at sub-locational level given the high density of population. The community unit, as defined in this context, comprises approximately 1 000 households or 5 000 people who live in the same geographical area, sharing resources and challenges. In most rural areas such a unit would be a sub-location, the lowest administrative unit. The CHWs in nomadic sites should be given more logistical support, given the distances that they must cover.

## List of abbreviations used

ANC: Antenatal care; CADs: Community action days; CBHIS: Community based health information system; CDDs: Community dialogue days; CHC: Community health committee; CHEW: Community health extension worker; CHS: Community Health Strategy; CHU: Community Health Units; CHW: Community health worker; CU: Community unit; DFATD: Foreign Affairs, Trade and Development Canada; DHMTs: District Health Management Teams; GHRI: Global Health Research Initiative; GLUK: Great Lakes University of Kisumu; HF: Health facility; HFD: Health facility delivery; HMIS: Health Management Information System; IDRC: International Development Research Centre; ITN: Insecticide treated net; MNH: Maternal and newborn health; MOPHS: Ministry of Public Health and Sanitation; MUAC: Mid-upper arm circumference; NHSSP II: National Health Sector Strategic Plan II; PI: Principal Investigator.

## Competing interests

The authors declare that they have no competing interests.

## Authors' contributions

All authors contributed equally and made substantial contributions to conception and design, acquisition of data, analysis and interpretation of data; drafting the article or revising it critically for important intellectual content.

## Declaration

The publication costs associated with this article are funded by Foreign Affairs, Trade and Development Canada and the International Development Research Centre through the Global Health Research Initiative.

This article has been published as part of *BMC Health Services Research* Volume 14 Supplement 1, 2014: Uptake and impact of research for evidence-based practice: lessons from the Africa Health Systems Initiative's research component. The full contents of the supplement are available online at http://www.biomedcentral.com/bmchealthservres/supplements/14/S1
